# Radiobiological Characterization of Canine Malignant Melanoma Cell Lines with Different Types of Ionizing Radiation and Efficacy Evaluation with Cytotoxic Agents

**DOI:** 10.3390/ijms20040841

**Published:** 2019-02-15

**Authors:** Hiroto Yoshikawa, Shigeaki Sunada, Hirokazu Hirakawa, Akira Fujimori, Suad Elmegerhi, Del Leary, Takamitsu A. Kato

**Affiliations:** 1 Department of Clinical Sciences, College of Veterinary Medicine, North Carolina State University, Raleigh, NC 27606, USA; hyoshik@ncsu.edu; 2 Department of Molecular Genetics, Medical Research Institute, Tokyo Medical and Dental University, Tokyo 113-8510, Japan; sunada.mgen@mri.tmd.ac.jp; 3 Department of Basic Medical Sciences for Radiation Damages, National Institute of Radiological Sciences, Chiba 263-8555, Japan; hirakawa.hirokazu@qst.go.jp (H.H.); fujimori.akira@qst.go.jp (A.F.); 4 Department of Environmental and Radiological Health Sciences, Colorado State University, Fort Collins, CO 80523, USA; hanoyara@rams.colostate.edu (S.E.); Del.Leary@colostate.edu (D.L.); 5 Cell Molecular Biology Program, Colorado State University, Fort Collins, CO 80523, USA

**Keywords:** canine malignant melanoma, ionizing radiation, photon, carbon ion, tumor hypoxia

## Abstract

Canine malignant melanoma (CMM) is a locally and systemically aggressive cancer that shares many biological and clinical characteristics with human mucosal melanoma. Hypofractionated radiation protocols have been used to treat CMM but little is known about its radiation biology. This pilot study is designed to investigate response of CMM cell lines to various ionizing radiations and cytotoxic agents to better understand this canine cancer. Four CMM cell lines were evaluated by clonogenic survival assay under aerobic and hypoxic conditions and parameters such as alpha beta (α/β) ratio, oxygen enhancement ratio (OER), and relative biological effectiveness (RBE) were calculated after ^137^Cs, 6 megavoltage (MV) photon, or carbon ion irradiation. Six cytotoxic agents (cisplatin, camptothecin, mitomycin C, bleomycin, methtyl methanesulfonate and etoposide) were also assessed for their efficacy. Under aerobic condition with 6 MV photon, the α/β ratio of the four cell lines ranged from 0.3 to >100, indicating a wide variation of cellular sensitivity. The ratio increased under hypoxic condition compared to aerobic condition and this was more dramatic in ^137^Cs and 6 MV photon treatments. OER of carbon was lower than ^137^Cs at D_10_ in 3 of the 4 cell lines. The RBE values generally increased with the increase of LET. Different cell lines showed sensitivity/resistance to different cytotoxic agents. This study revealed that CMM has a wide range of radiosensitivity and that hypoxia can reduce it, indicating that widely used hypofractionated protocols may not be optimal for all CMM patients. Several cytotoxic agents that have never been clinically assessed can improve treatment outcome.

## 1. Introduction

Canine malignant melanoma (CMM) is a common oral and cutaneous tumor in dogs, and recently CMMs have been considered as a large animal model for human mucosal melanomas [1]. Previous studies revealed several prognostic factors for clinical outcome following treatment interventions such as tumor location within the mouth, tumor size, and completeness of histopathological margin [2,3]. CMM is highly invasive locally and carries a high tendency to spread regionally and systemically, yielding significantly shorter survival times [4]. Numerous studies have failed to develop a more effective local and systemic treatment regimen to improve survival times [2], and currently surgical excision with adjuvant local and/or systemic therapy is considered as the standard of care. Radiation therapy is a commonly used locoregional treatment for CMMs [3,5,6,7,8]. Different radiation therapy protocols have been used in veterinary radiation therapy. In a previously reported study that evaluated clinical outcomes after different radiation therapy protocols to treat CMM, no significant advantage in treatment outcome was found in dogs that were treated with a fractionated protocol compared to those that were treated with a hypofractionated protocol. [7]. This report may be part of the reason why hypofractionated radiation protocols with a large dose per fraction are most commonly used to treat canine CMM [8,9,10]. Other reasons may include that the hypofractionated radiation protocol is generally well tolerated by the canine patients and in veterinary radiation oncology, patients need to be under general anesthesia or sedation to immobilize them, therefore having fewer treatment sessions is more practical and convenient. However, complete response cannot be achieved in all clinical cases and in fact, large tumor volume was a part of a joint prognostic factor in the aforementioned study. The background biology of this variation in treatment outcome has not been well studied. Parameters that help understand a cell’s radiobiology include alpha beta ratio, survival fraction (SF) at a specific radiation dose (i.e., SF at 2 Gy; SF_2_), and radiation dose that results in a specific cell survival rate (i.e., 10% survival; D_10_). Knowing those parameters may help to better understand radiobiology of CMM and create a more effective radiation therapy protocol.

There are several different types of ionizing radiation that are currently used to treat human and veterinary cancer patients, although the most common is megavoltage photon beam radiation delivered with linear accelerators. However, more recently over the last few decades, heavy ion radiation facilities for research and clinical purposes have been constructed, equipped with a capability to accelerate heavier particles such as protons, and heavy ions such as carbon and iron nuclei. The heavy ion beams have a unique physical characteristic that is called Bragg Peak that where the dose ramps up quickly and locally deep within the tissue. The depth can be controlled and is determined by the initial energy of the ion and this peak can be also spread out to cover a wide area by delivering a range of energies, known as the spread out Bragg peak (SOBP). Importantly, as the ion slows the radiation deposited can be very dense at the end of the SOBP, increasing the level of linear energy transfer (LET) and potentially the relative biological effectiveness (RBE). By positioning the spread out Bragg peak (SOBP) at the target lesion, a very high dose can be delivered to the target lesion while sparing the distal tissues and minimizing proximal tissues since the ions have little interaction before the Bragg peak. Moreover, heavy ion beams such as carbon and iron ion create very prominent Bragg peaks, and are more efficient in cell damaging and are less dependent on tissue microenvironment oxygen level unlike photon beams [11]. Because of those biological and physical advantages, heavy ion beams are used in biology research and in human cancer treatment [12,13,14], though the availability is limited.

Since CMM is highly metastatic disease and many clinical patients eventually develop systemic spread such as lung metastasis, chemotherapeutic drugs also have been used to treat CMMs. Carboplatin and nonsteroidal anti-inflammatory drugs especially piroxicam are the two most commonly used drugs in veterinary practices. Their efficacy for local tumor control as well as for preventing development of systemic progression has been clinically studied with conflicting conclusions [7,8,15,16]. Other chemotherapeutic drugs that were previously evaluated to treat CMMs include toceranib phosphate, temozolomide, doxorubicin, dacarbazine, lomustine, cyclophosphamide, cisplatin, and mitoxantrone but their efficacy is still controversial [9,10,15,17,18,19]. For human melanomas of different origin, various chemotherapeutic drugs including Camptothecin [20,21,22,23,24], mitomycin C [25,26,27], bleomycin [28,29], and etoposide [30] have been evaluated in vitro and/or in vivo. To the authors’ knowledge, there are no studies that investigated efficacy of those agents against CMMs in veterinary oncology at the time of writing.

This pilot study was designed to characterize four established CMM cell lines by using different types of ionizing radiations and cytotoxic agents to deepen our understanding about CMMs and eventually assist the creation of a more effective multi-modality treatment regimen.

## 2. Results

### 2.1. Survival Curves

**Figure 1:**
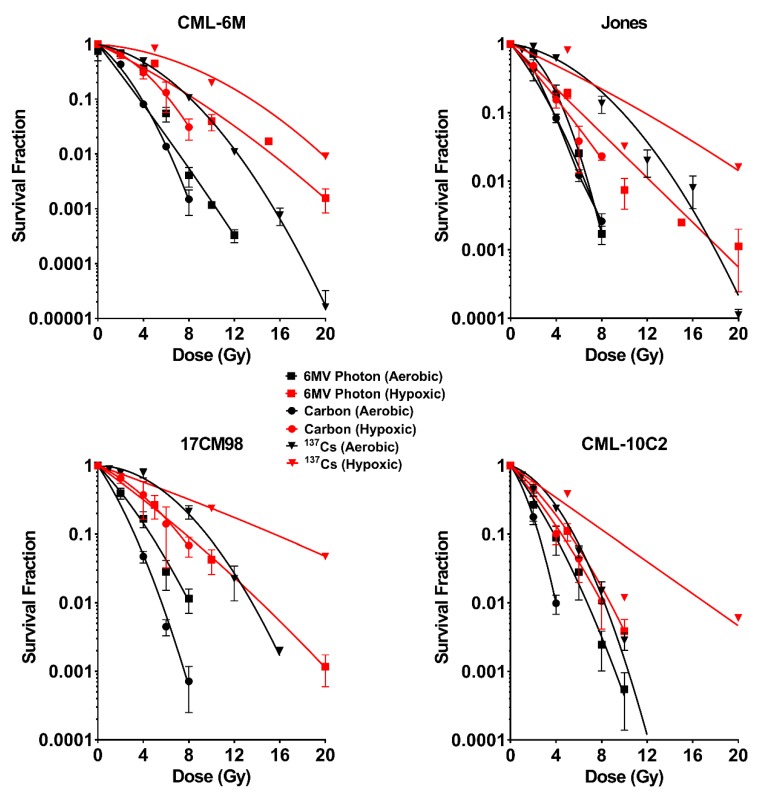
Results of clonogenic survival assay performed with ^137^Cs, 6MV photon, and carbon-ion radiation under aerobic (black) and hypoxic (red) conditions. Linear-quadratic cell survival curves were fitted and used to calculate radiobiologic parameters. Error bars indicate standard error of the means from at least three independent experiments.

**Figure 2:**
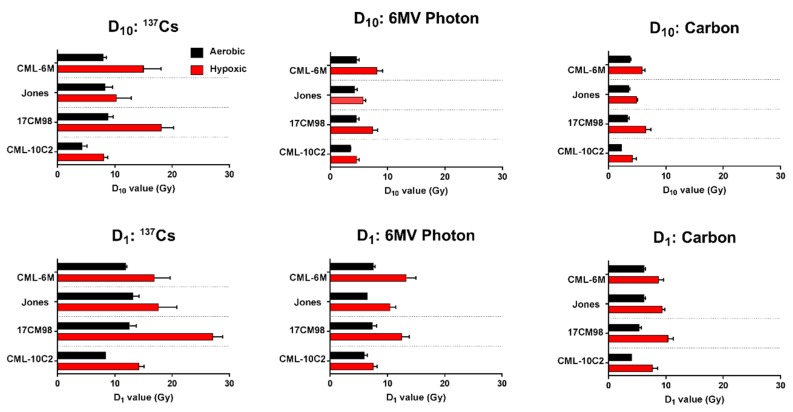
Summary of D_10_ and D_1_, which are radiation dose to kill 90% and 99% of cells, respectively, using four canine malignant melanoma cell lines irradiated with ^137^Cs, 6MV photon, or carbon ion radiation under aerobic (black) and hypoxic (red) conditions. Error bars indicate standard error of the means from at least three independent experiments.

Cell survival curves generated from colony formation assays for each cell line and each beam quality are shown in Figure 1. Cell survival was highest in all cell lines for the lowest LET, ^137^Cs, treatment, followed by 6MV photon then carbon. All cell lines became more resistant to radiation under hypoxic condition, compared to under aerobic condition. This was most notable for the lower LET beam qualities (^137^Cs and 6MV). The change in radiosensitivity between aerobic and hypoxic conditions were minimal with carbon ion irradiation compared to ^137^Cs and 6MV photon beams. These findings can be quantified by D_10_, D_1_, SF_2_, and SF_8_, all interpolated from the survival curve. (Table 1 and Figure 2). When compared between cell lines, CML-10C2 were the most radiosensitive cell line while other three cell lines were more radioresistant in a similar degree. The difference in radiosensitivity between cell lines was subjectively more prominent with ^137^Cs compared to carbon. For ^137^Cs, RBE values under hypoxic condition were unavailable in Jones and 17CM98 cell lines at D_1_ since D_1_ values were not calculable from the linear quadratic model graphs.

### 2.2. α/β Ratio

α/β ratio was calculated for aerobic and hypoxic conditions. There were noticeable variations in α/β ratios when comparing different cell lines as well as between aerobic and hypoxic conditions within each cell line (Table 2). In general, the α/β ratio increased under hypoxic conditions compared to under aerobic conditions and this change was more dramatic in ^137^Cs and 6MV photon treatments.

### 2.3. Oxygen Enhancement Ratio

**Figure 3:**
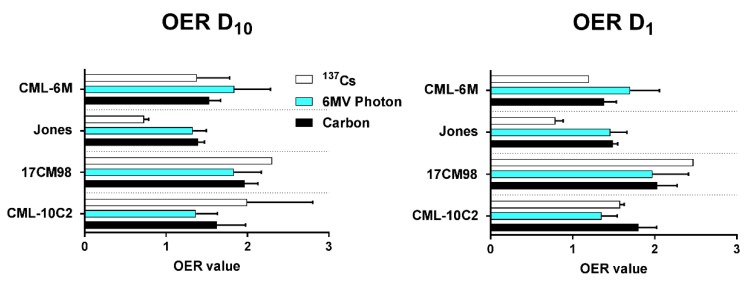
Summary of mean oxygen enhancement ratio of four canine malignant melanoma cell lines irradiated with ^137^Cs (white), 6MV photon (blue), or carbon ion (black) radiation. Error bars indicate standard error of the means from at least three independent experiments.

Mean oxygen enhancement ratio ranged from 1.3 to 1.9 for 6MV photon, from 1.3 to 2.0 for carbon, and from 0.7 to 2.3 for ^137^Cs (Figure 3) at D_10_ and D_1_.

### 2.4. Relative Biological Effectiveness

**Figure 4:**
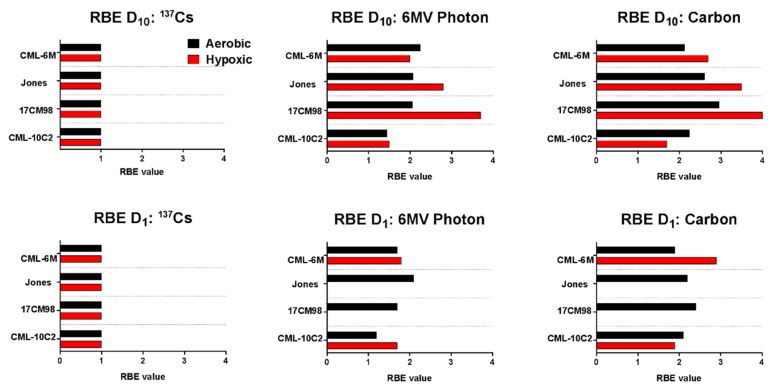
Summary of relative biological effectiveness of four canine malignant melanoma cell lines irradiated with ^137^Cs, 6MV photon, or carbon ion radiation, under aerobic (black) and hypoxic (red) conditions. The values presented here are mean of at least three independent experiments.

The RBE values obtained under aerobic and hypoxic conditions ranged from 1.2 to 2.3 and 1.5 to 3.7 for 6MV photon, and from 1.9 to 3.0 and 1.7 to 4.0 for Carbon ion, respectively (Figure 4). In general, RBE values increased as LET increased.

### 2.5. Drug Sensitivity Test

**Figure 5:**
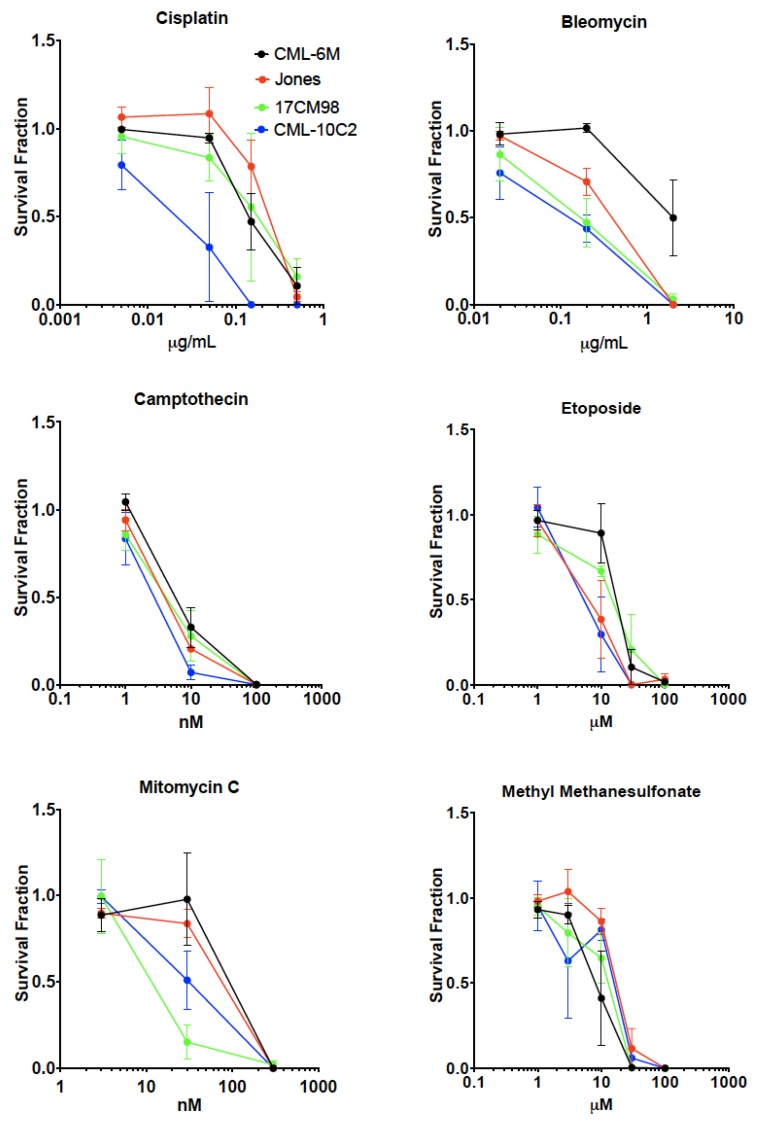
Summary of drug sensitivity tests of four canine malignant melanoma cell lines with different cytotoxic agents. Error bars indicate standard error of the means from at least three independent experiments.

Results of drug sensitivity tests with cisplatin, bleomycin, camptothecin, etoposide, methyl methanesulfonate and mitomycin C were summarized in Figure 5. In general, survival fraction decreased in a dose dependent manner in all cell lines and with all treatments. CML-10C2 showed increased sensitivity to all drugs, making it the most chemosensitive cell line among the evaluated four cell lines. 17CM98 cells showed increased sensitivity to bleomycin and mitomycin C. CML-6M showed relative resistance to all tested drugs. Jones showed increased sensitivity to bleomycin and etoposide.

## 3. Discussion

External beam radiation therapy is commonly used to treat CMMs especially in non-surgical cases or after incomplete surgical excision. Historically those tumors have been treated with hypofractionated protocols with a larger dose per fraction [5,6,7,31]. Even though the hypofractionated protocol is clinically more convenient and widely used (i.e., fewer anesthetic episodes, less expensive compared to conventional fractionated protocols), no study has assessed radiobiology of CMMs, therefore the rational of choosing the hypofractionated protocol is unclear. Our current study indicated that the CMM cell lines we evaluated have a wide range of the alpha-beta ratio, which is one of the indicators of radiation sensitivity of the tested cells. With 6MV photon, which is the widely used ionizing radiation in the veterinary clinical radiotherapy, the alpha beta ratio ranged from smaller than 1 to larger than 100. This may indicate that many patients experienced local treatment failure after hypofractionated radiotherapy received suboptimal protocol [3]. A future study to correlate pattern/rate of local treatment failure with radiotherapy regimen or biological indices (i.e., mitotic index) may help us choose the most effective radiotherapy protocol for each patient.

Interestingly, the alpha beta ratio increased in most of the evaluated CMM cell lines under hypoxic condition. This finding indicates that the efficacy of ionizing radiation to kill CMM cells can be dramatically influenced by the oxygen level in the tumor tissue. The OERs in the current study were generally higher with ^137^Cs than with 6MV photon and the OERs with carbon ion tends to be the lowest. This suggests that carbon ion radiation therapy is more efficient to kill the CMM cancer cells regardless of the oxygen level in the tumor. The RBE is used to compare test radiation’s biological effect (clonogenic death) with that of a reference radiation (^137^Cs). Unsurprisingly, both 6MV photon and carbon ion radiation showed RBE higher than 1 (^137^Cs) under both aerobic and hypoxic conditions. Carbon ion showed higher RBE than 6MV photon. Based on those findings, carbon ion radiotherapy is more efficient in leading CMM cells into clonogenic death and its efficacy is dependent on tumor oxygenation status. This may also indicate that carbon ion radiotherapy may allow veterinary radiation oncologists to treat dogs with CMMs with even fewer fractionations to achieve the same or better locoregional tumor control, thanks to its higher biological efficacy. A future clinical study to validate this finding in vivo is required.

Cytotoxic agents are commonly used along with nonsteroidal anti-inflammatory drugs, surgery, radiotherapy, or combination of those. Cisplatin, Bleomycin, camptothecin, etoposide, methyl methanesulfonate, and mitomycin C were evaluated in-vivo and in-vitro manners to assess their potential clinical efficacy to treat various canine malignancies [15,31,32,33,34,35,36,37,38,39,40,41]. Among them, cisplatin is the most studied cytotoxic agent to treat CMMs. Clinical treatment outcome of CMMs is still not great, however, due to either local treatment failure and/or systemic spread, making the role of the systemic treatment with the currently-utilized cytotoxic agents anecdotal. The current study evaluated efficacy of cytotoxic agents against CMM cell lines with a hope to identify a new candidate agent for further clinical investigation. As was shown in previous studies, our current data supported its cytotoxic activity. Bleomycin, camptothecin, etoposide, and mitomycin C have never been evaluated for their clinical benefit against CMMs. Our study suggests that those cytotoxic agents, especially camptothecin (Topoisomerase I inhibitor) and etoposide (Topoisomerase II inhibitor), may be of worth assessing their clinical benefit. The current study may serve as a starting point for a future in-vivo evaluation of those cytotoxic agents for canine malignant melanomas. Evaluation of combination effects between carbon ion irradiation and topoisomerase inhibitor may be an important study for CMM cell lines.

## 4. Materials and Methods

### 4.1. Cell Lines

Four canine oral melanoma cell lines (Jones, 17CM98, CML-6M, and CML-10C2) were generously supplied by Dr. Douglas Thamm (Colorado State University, Fort Collins, CO, USA) and grown in Minimum Essential Medium (MEM/EBSS, Thermo Fisher Scientific, Waltham, MA, USA) supplemented with 10% heat inactivated fetal bovine serum (FBS: Sigma-Aldrich, St Louis, MO, USA), 1% MEM vitamins, non-essential amino acids, sodium pyruvate, penicillin, streptomycin, and Fungizone. Those cell lines were previously characterized in previously published literature [42,43,44].

### 4.2. Hypoxic Treatment

The hypoxic condition was created using a commercially available oxygen absorber-CO_2_ generator placed in a dedicated container by following manufacture’s protocol (Anaeropack Kenki 5%, Mitsubishi Gas Chemical Company, INC., Tokyo, Japan). The oxygen-level was then monitored by placing an indicator within the container (Anaero-indicator, Mitsubishi Gas Chemical Company, INC.) [45].

### 4.3. Irradiation

Photon irradiation: Photon irradiation was performed at room temperature by using a clinical linear accelerator (Varian Trilogy, Varian Medical Systems, Inc., Palo Alto, CA, USA) located at the Colorado State University Flint Animal Cancer Center. Only 6MV photon beams (6 Gy/min) from a single 30 × 30 cm field size was used. The dose was uniform and flat within the area where the cells were placed. The cells sat at the bottom of the flasks with 2 mm of media on top and 10 cm of solid water for appropriate backscatter. This allowed the dose to be corrected to 2 mm percent depth dose at 100 cm source to surface distance, which are common setup parameters for absolute calibration and therefore dose could be easily calculated from standard scattering tables.

Carbon-ion irradiation: Carbon-ion irradiation experiments were carried out at the National Institute of Radiological Sciences (NIRS) in Chiba, Japan. The details concerning the beam characteristics of the carbon radiation, biological irradiation procedures, and dosimetry have been described elsewhere [46,47,48]. Briefly, carbon ions were accelerated at 290 MeV/nucleon of initial energy and spread out with a ridge filter creating a SOBP over 6 cm. The monolayer cell culture was irradiated at the center (50 keV/μm of average LET) of the SOBP at a distance of 119 mm from the entrance (1 Gy/min) [49].

^137^Cs irradiation: Gamma-ray irradiation was performed at room temperature by using a ^137^Cs gamma-rays irradiator located at Colorado State University (J.L. Shepherd Model Mark I-68 nominal 6000Ci, J.L. Shepherd, Carlsbad, CA, USA) with 2.5 Gy/min of dose rate.

### 4.4. Colony Formation Assay

Trypsinized cells were plated on P-60 dishes to obtain approximately 100 colonies per dish. Based on plating efficiency and radiosensitivity of each cell line, plated number of cells were varied (200-200000 cells). Plated cells were treated under normal aerobic condition or hypoxic condition before irradiation. After irradiation, cells were cultured for 7-day incubation period to form colonies, without changing cell culture media, cells were washed in 0.9% sodium chloride and fixed in 100% ethanol, then stained with 0.1% crystal violet dye (Sigma-Aldrich). Lastly, with microscopic analysis, colonies containing more than 50 cells were scored as survivors. The cell survival fraction was determined as the ratio of colony of irradiated cells to that of non-irradiated cells.

### 4.5. Drug Sensitivity Study

Trypsinized cells were plated on P-60 dishes to obtain approximately 100 colonies per dish. Based on plating efficiency and drug-sensitivity of each cell line, plated number of cells were varied (200-20000 cells). Once cells were attached, cells were treated with various dosages of cisplatin (DNA crosslinker), bleomycin (DNA double strand break inducer), etoposide (Topoisomerase II inhibitor), camptothecin (Topoisomerase I inhibitor), and mitomycin C (alkylating agent, free radical formation, DNA crosslinker), Methyl methanesulfonate (alkylating agent) during entire colony formation period for 7 days. After a 7-day incubation period, cells were washed in 0.9% sodium chloride and fixed in 100% ethanol, then stained with 0.1% crystal violet dye (Sigma) for colony formation analysis.

### 4.6. Data Analyses

All experiments were carried out at least three times independently. Data handling and analyses were performed by using a commercially available software (GraphPad Prism version 7.00, GraphPad Software, La Jolla, CA, USA, www.graphpad.com).

Survival curves were generated by applying linear quadratic regression model. Survival fraction (SF) was obtained by interpolating the linear quadratic survival curves. In addition to the standard SF_2_ (survival fraction at 2 Gy), survival fraction at 8 Gy, a commonly used dose in clinical radiotherapy for canine oral malignant melanomas was also recorded and reported (SF_8_). α/β ratio was calculated by the following equation:

α/β = exp[−(αD + βD^2^)] (1)

where D is a radiation dose (Gy).

From this regression curves, D_10_ and D_1_ values were obtained by GraphPad Prism software.

Oxygen enhancement ratio (OER) was calculated for each radiation type by the following equation:

OER = Dose (Gy) under hypoxic condition/Dose (Gy) under aerobic condition(2)

Relative biological effectiveness (RBE) was calculated by the following equation:

RBE = Dose (Gy)(^137^Cs)/Dose (Gy) (6MV photon or Carbon) (3)

For OER and RBE, both D_10_ and D_1_ (radiation dose that kills 90 % and 99 % of cells, respectively) were used as an endpoint.

## 5. Conclusions

In conclusion, the current study showed that CMMs may not be uniformly sensitive to radiotherapy with a large fraction size and their radiosensitivity may be dramatically impacted by local oxygenation status. High LET radiotherapy such as carbon ion may possess a biological and clinical benefit from more effective cell killing, allowing veterinary radiation oncologists to treat with even less fractionation. Also, some additional cytotoxic agents showed potential clinical benefit, which may be worth evaluating in a clinical study.

## Figures and Tables

**Table 1:** Summary of SF_2_ and SF_8_, survival fraction at 2 and 8 Gy irradiation of four canine malignant melanoma cell lines irradiated with ^137^Cs, 6MV photon, or carbon ion radiation under aerobic and hypoxic conditions. Values are mean of at least three independent experiments.

			SF_2_ and SF_8_			
			CML-6M	Jones	17CM98	CML-10C2
SF_2_	^137^Cs	Aerobic	0.75	0.84	0.89	0.63
		Hypoxic	0.85	0.77	0.85	0.55
	6MV Photon	Aerobic	0.29	0.65	0.41	0.33
		Hypoxic	0.62	0.47	0.57	0.48
	Carbon	Aerobic	0.37	0.33	0.27	0.18
		Hypoxic	0.66	0.41	0.65	0.39
SF_8_	^137^Cs	Aerobic	0.11	0.20	0.20	0.01
		Hypoxic	0.44	0.35	0.53	0.09
	6MV Photon	Aerobic	0.01	0.002	0.01	0.003
		Hypoxic	0.12	0.05	0.09	0.02
	Carbon	Aerobic	0.001	0.002	0.001	<0.001
		Hypoxic	0.03	0.02	0.07	0.01

**Table 2:** Summary of alpha-beta ratio of four canine malignant melanoma cell lines irradiated with ^137^Cs, 6MV photon, or carbon ion radiation under aerobic and hypoxic conditions. The values presented here are from interpolation of linear-quadratic cell survival curves using mean values of at least three independent experiments.

		α/β ratio			
		CML-6M	Jones	17CM98	CML-10C2
^137^Cs	Aerobic	4.3	2.9	0.3	2.4
	Hypoxic	22.7	>100	>100	>100
6MV Photon	Aerobic	>100	0.3	19.2	17.9
	Hypoxic	>100	47.8	83.3	14.1
Carbon	Aerobic	7.4	13.8	13.1	3.9
	Hypoxic	3.6	77.9	8.4	27.5

## Abbreviations

CMM	Canine malignant melanoma
OER	Oxygen enhancement ratio
RBE	Relative biological effectiveness
SF	Survival fraction
SOBP	Spread out Bragg peak
LET	Linear energy transfer

## References

[1] Hernandez B., Adissu H.A., Wei B.R., Michael H.T., Merlino G., Simpson R.M. (2018). Naturally Occurring Canine Melanoma as a Predictive Comparative Oncology Model for Human Mucosal and Other Triple Wild-Type Melanomas. Int. J. Mol. Sci..

[2] Klein M.K. (2003). Multimodality therapy for head and neck cancer. Vet. Clin. North Am. Small Anim. Pract..

[3] Theon A.P., Rodriguez C., Madewell B.R. (1997). Analysis of prognostic factors and patterns of failure in dogs with malignant oral tumors treated with megavoltage irradiation. J. Am. Vet. Med. Assoc..

[4] Tuohy J.L., Selmic L.E., Worley D.R., Ehrhart N.P., Withrow S.J. (2014). Outcome following curative-intent surgery for oral melanoma in dogs: 70 cases (1998–2011). J. Am. Vet. Med. Assoc..

[5] Bateman K.E., Catton P.A., Pennock P.W., Kruth S.A. (1994). 0-7-21 radiation therapy for the treatment of canine oral melanoma. J. Vet. Intern. Med..

[6] Blackwood L., Dobson J.M. (1996). Radiotherapy of oral malignant melanomas in dogs. J. Am. Vet. Med. Assoc..

[7] Proulx D.R., Ruslander D.M., Dodge R.K., Hauck M.L., Williams L.E., Horn B., Price G.S., Thrall D.E. (2003). A retrospective analysis of 140 dogs with oral melanoma treated with external beam radiation. Vet. Radiol. Ultrasound..

[8] Murphy S., Hayes A.M., Blackwood L., Maglennon G., Pattinson H., Sparkes A.H. (2005). Oral malignant melanoma - the effect of coarse fractionation radiotherapy alone or with adjuvant carboplatin therapy. Vet. Comp. Oncol..

[9] Boston S.E., Lu X., Culp W.T., Montinaro V., Romanelli G., Dudley R.M., Liptak J.M., Mestrinho L.A., Buracco P. (2014). Efficacy of systemic adjuvant therapies administered to dogs after excision of oral malignant melanomas: 151 cases (2001–2012). J. Am. Vet. Med. Assoc..

[10] Cancedda S., Rohrer Bley C., Aresu L., Dacasto M., Leone V.F., Pizzoni S., Gracis M., Marconato L. (2016). Efficacy and side effects of radiation therapy in comparison with radiation therapy and temozolomide in the treatment of measurable canine malignant melanoma. Vet. Comp. Oncol..

[11] Hall E., Giaccia A. (2018). Linear energy transfer and relative biologic effectiveness. Radiobiology for the Radiologist.

[12] Hayashi K., Koto M., Demizu Y., Saitoh J.I., Suefuji H., Okimoto T., Ohno T., Shioyama Y., Takagi R., Ikawa H. (2018). A retrospective multicenter study of carbon-ion radiotherapy for external auditory canal and middle ear carcinomas. Cancer Med..

[13] Takagi M., Demizu Y., Nagano F., Terashima K., Fujii O., Jin D., Mima M., Niwa Y., Katsui K., Suga M. (2018). Treatment outcomes of proton or carbon ion therapy for skull base chordoma: a retrospective study. Radiat. Oncol..

[14] Abe T., Ohno T., Koto M., Demizu Y., Suefuji H., Tsuji H., Okimoto T., Shioyama Y., Saitoh J.I., Shirai K. (2018). A multi-institutional retrospective study of carbon-ion radiotherapy for non-squamous cell malignant tumors of the nasopharynx: Subanalysis of Japan Carbon-Ion Radiation Oncology Study Group study 1402 HN. Cancer Med..

[15] Boria P.A., Murry D.J., Bennett P.F., Glickman N.W., Snyder P.W., Merkel B.L., Schlittler D.L., Mutsaers A.J., Thomas R.M., Knapp D.W. (2004). Evaluation of cisplatin combined with piroxicam for the treatment of oral malignant melanoma and oral squamous cell carcinoma in dogs. J. Am. Vet. Med. Assoc..

[16] Rassnick K.M., Ruslander D.M., Cotter S.M., Al-Sarraf R., Bruyette D.S., Gamblin R.M., Meleo K.A., Moore A.S. (2001). Use of carboplatin for treatment of dogs with malignant melanoma: 27 cases (1989–2000). J. Am. Vet. Med. Assoc..

[17] Wouda R.M., Hocker S.E., Higginbotham M.L. (2018). Safety evaluation of combination carboplatin and toceranib phosphate (Palladia) in tumour-bearing dogs: A phase I dose finding study. Vet. Comp. Oncol..

[18] Choisunirachon N., Jaroensong T., Yoshida K., Saeki K., Mochizuki M., Nishimura R., Sasaki N., Nakagawa T. (2015). Effects of low-dose cyclophosphamide with piroxicam on tumour neovascularization in a canine oral malignant melanoma-xenografted mouse model. Vet. Comp. Oncol..

[19] Ogilvie G.K., Obradovich J.E., Elmslie R.E., Vail D.M., Moore A.S., Straw R.C., Dickinson K., Cooper M.F., Withrow S.J. (1991). Efficacy of mitoxantrone against various neoplasms in dogs. J. Am. Vet. Med. Assoc..

[20] Cichorek M. (2011). Camptothecin-induced death of amelanotic and melanotic melanoma cells in different phases of cell cycle. Neoplasma.

[21] Liu X.P., Zhou S.T., Li X.Y., Chen X.C., Zhao X., Qian Z.Y., Zhou L.N., Li Z.Y., Wang Y.M., Zhong Q. (2010). Anti-tumor activity of N-trimethyl chitosan-encapsulated camptothecin in a mouse melanoma model. J. Exp. Clin. Cancer Res..

[22] Loch-Neckel G., Nemen D., Puhl A.C., Fernandes D., Stimamiglio M.A., Alvarez Silva M., Hangai M., Santos Silva M.C., Lemos-Senna E. (2007). Stealth and non-stealth nanocapsules containing camptothecin: in-vitro and in-vivo activity on B16-F10 melanoma. J. Pharm. Pharmacol..

[23] Dora C.L., Alvarez-Silva M., Trentin A.G., de Faria T.J., Fernandes D., da Costa R., Stimamiglio M., Lemos-Senna E. (2006). Evaluation of antimetastatic activity and systemic toxicity of camptothecin-loaded microspheres in mice injected with B16-F10 melanoma cells. J. Pharm. Pharm. Sci..

[24] Gottlieb J.A., Luce J.K. (1972). Treatment of malignant melanoma with camptothecin (NSC-100880). Cancer Chemother. Rep..

[25] Li S., Au W.W., Schmoyer R.L., Hsu T.C. (1982). Baseline and mitomycin-C-induced sister chromatic exchanges ina melanoma and a colon tumor cell line. Cancer Genet. Cytogenet..

[26] Pedersen J.E., Barron G. (1984). Radiation and concurrent chemotherapy for the treatment of Lewis lung tumor and B16 melanoma tumor in C57/BL mice. Int. J. Radiat. Oncol. Biol. Phys..

[27] Creagan E.T., Edmonson J.H., Ahmann D.L., Chang M. (1985). Phase II study of mitomycin in disseminated malignant melanoma. Cancer Treat Rep..

[28] Nathanson L., Wittenberg B.K. (1980). Pilot study of vinblastine and bleomycin combinations in the treatment of metastatic melanoma. Cancer Treat Rep..

[29] Nathanson L., Kaufman S.D., Carey R.W. (1981). Vinblastine, infusion, bleomycin, and cis-dichlorodiammine-platinum chemotherapy in metastatic melanoma. Cancer.

[30] Bajetta E., Buzzoni R., Viviani S., Vaglini M., Nava M., Bonadonna G. (1985). Prospective randomized trial in advanced malignant melanoma with cis-platinum, vindesine, and etoposide vs. cis-platinum, vindesine, and lomustine. Am. J. Clin. Oncol..

[31] Freeman K.P., Hahn K.A., Harris F.D., King G.K. (2003). Treatment of dogs with oral melanoma by hypofractionated radiation therapy and platinum-based chemotherapy (1987–1997). J. Vet. Intern. Med..

[32] Milevoj N., Tratar U.L., Nemec A., Brozic A., Znidar K., Sersa G., Cemazar M., Tozon N. (2018). A combination of electrochemotherapy, gene electrotransfer of plasmid encoding canine IL-12 and cytoreductive surgery in the treatment of canine oral malignant melanoma. Res. Vet. Sci..

[33] Batschinski K., Dervisis N., Kitchell B., Newman R., Erfourth T. (2018). Combination of Bleomycin and Cytosine Arabinoside Chemotherapy for Relapsed Canine Lymphoma. J. Am. Anim. Hosp. Assoc..

[34] Han S.C., Kim D.G., Han E.H., Kim Y.B., Hwang I.C., Kim C.Y. (2010). Toxicity study of a new camptothecin anti-cancer agent CKD-602 in dogs: 4-week continuous intravenous dose by infusion pump and 4-week repeated intravenous dose. Regul. Toxicol. Pharmacol..

[35] Boye P., Serres F., Marescaux L., Hordeaux J., Bouchaert E., Gomes B., Tierny D. (2017). Dose escalation study to evaluate safety, tolerability and efficacy of intravenous etoposide phosphate administration in 27 dogs with multicentric lymphoma. PLoS ONE.

[36] Flory A.B., Rassnick K.M., Balkman C.E., Kiselow M.A., Autio K., Beaulieu B.B., Lewis L.D. (2008). Oral bioavailability of etoposide after administration of a single dose to tumor-bearing dogs. Am. J. Vet. Res..

[37] Abbo A.H., Jones D.R., Masters A.R., Stewart J.C., Fourez L., Knapp D.W. (2010). Phase I clinical trial and pharmacokinetics of intravesical mitomycin C in dogs with localized transitional cell carcinoma of the urinary bladder. J. Vet. Intern. Med..

[38] Shapiro W., Kitchell B.E., Fossum T.W., Couto C.G., Theilen G. (1988). Cisplatin for treatment of transitional cell and squamous cell carcinomas in dogs. J. Am. Vet. Med. Assoc..

[39] Shapiro W., Fossum T.W., Kitchell B.E., Couto C.G., Theilen G.H. (1988). Use of cisplatin for treatment of appendicular osteosarcoma in dogs. J. Am. Vet. Med. Assoc..

[40] Knapp D.W., Richardson R.C., Bonney P.L., Hahn K. (1988). Cisplatin therapy in 41 dogs with malignant tumors. J. Vet. Intern. Med..

[41] Kitchell B.E., Brown D.M., Luck E.E., Woods L.L., Orenberg E.K., Bloch D.A. (1994). Intralesional implant for treatment of primary oral malignant melanoma in dogs. J. Am. Vet. Med. Assoc..

[42] Maeda J., Froning C.E., Brents C.A., Rose B.J., Thamm D.H., Kato T.A. (2016). Intrinsic Radiosensitivity and Cellular Characterization of 27 Canine Cancer Cell Lines. PLoS ONE.

[43] Wolfe L.G., Oliver J.L., Smith B.B., Toivio-Kinnucan M.A., Powers R.D., Brawner W.R., Henderson R.A., Hankes G.H. (1987). Biologic characterization of canine melanoma cell lines. Am. J. Vet. Res..

[44] Fowles J.S., Dailey D.D., Gustafson D.L., Thamm D.H., Duval D.L. (2017). The Flint Animal Cancer Center (FACC) Canine Tumour Cell Line Panel: a resource for veterinary drug discovery, comparative oncology and translational medicine. Vet. Comp. Oncol..

[45] Cartwright I.M., Su C., Haskins J.S., Salinas V.A., Sunada S., Yu H., Uesaka M., Hirakawa H., Chen D.J., Fujimori A. (2018). DNA Repair Deficient Chinese Hamster Ovary Cells Exhibiting Differential Sensitivity to Charged Particle Radiation under Aerobic and Hypoxic Conditions. Int. J. Mol. Sci..

[46] Suzuki M., Kase Y., Yamaguchi H., Kanai T., Ando K. (2000). Relative biological effectiveness for cell-killing effect on various human cell lines irradiated with heavy-ion medical accelerator in Chiba (HIMAC) carbon-ion beams. Int. J. Radiat. Oncol. Biol. Phys..

[47] Kamada T., Tsujii H., Tsuji H., Yanagi T., Mizoe J.E., Miyamoto T., Kato H., Yamada S., Morita S., Yoshikawa K. (2002). Efficacy and safety of carbon ion radiotherapy in bone and soft tissue sarcomas. J. Clin. Oncol..

[48] Cartwright I.M., Bell J.J., Maeda J., Genet M.D., Romero A., Fujii Y., Fujimori A., Kitamuta H., Kamada T., Chen D.J. (2015). Effects of targeted phosphorylation site mutations in the DNA-PKcs phosphorylation domain on low and high LET radiation sensitivity. Oncol. Lett..

[49] McMillan D.D., Maeda J., Bell J.J., Genet M.D., Phoonswadi G., Mann K.A., Kraft S.L., Kitamura H., Fujimori A., Yoshii Y. (2015). Validation of 64Cu-ATSM damaging DNA via high-LET Auger electron emission. J. Radiat. Res..

